# Comparison of Vascular Morphometry in Jawbones and Long Bones: Micro-CT Study in a Rat Model Treated with Zoledronic Acid

**DOI:** 10.1155/2021/6651318

**Published:** 2021-05-12

**Authors:** Jing Yi Wang, Ru Qing Yu, Lei Huo, Nian Jing Rao, Weijia William Lu, Li Wu Zheng

**Affiliations:** ^1^Discipline of Oral and Maxillofacial Surgery, Faculty of Dentistry, The University of Hong Kong, Prince Philip Dental Hospital, Hong Kong; ^2^Department of Orthodontics, The First Affiliated Hospital of Zhengzhou University, Zhengzhou, Henan Province, China; ^3^Department of Orthopedics and Traumatology, Li Ka Shing Faculty of Medicine, The University of Hong Kong, Hong Kong; ^4^Qiongtai Normal University, Hainan, China

## Abstract

The study was aimed at investigating the effect of zoledronic acid on vascular morphometry in jawbones and long bones on a rat model. Twenty-four skeletal mature Sprague-Dawley female rats were administered oncologic dose of zoledronic acid (ZA) or normal saline for 4 weeks and then subjected to tooth extraction on the mandible and maxilla and a bone defect creation on the femur. After the surgical procedures, ZA or saline treatment was continued until sacrifice at week 2, week 4, and week 8 postoperatively. Vascular perfusion with MICROFIL was performed on all the animals. Micro-CT analysis demonstrated a tendency of decreased vessel density and vessel number in ZA-treated groups but no statistical difference. In conclusion, the neovessel formation is suppressed but not significantly by ZA treatment, indicating that angiogenesis inhibition may contribute to the development of MRONJ but does not play a key role.

## 1. Introduction

Bisphosphonates (BPs) are widely used to manage bone loss in postmenopausal or aged patients and to prevent and reduce cancer-related bone complications. Despite the great clinical benefits, a severe complication, medication-related osteonecrosis of the jaw (MRONJ), has been reported. The large number of patients under BP treatment for various medical indications and the poorly understood etiology and pathophysiology of BRONJ render a serious concern of clinicians and society.

Despite the large number of clinical and basic science studies, the underlying mechanisms of MRONJ remain poorly understood. Although most studies considered the BP-induced remodeling suppression to be a key player in MRONJ (Allen et al., 2011), it is believed that impaired angiogenesis also contribute to the development of BRONJ while almost all the biopsy samples from patients with MRONJ showed avascular necrosis (*Allen* et al. 2009). Many studies have reported the inhibitory effect of BPs on tumor angiogenesis (*Reusser* et al.*, 2014;* [1]), which is a favorable effect for cancer treatment yet a potential cause of avascular bone necrosis. Several studies showed that BP treatment significantly decreased vascular endothelial growth factor (VEGF) circulating level in cancer patients with bone metastases [2, 3] [4]. However, the effect of BPs on vascularization, particularly the site-specific effects of BPs on neovessel formation in jawbones and long bones, has not been fully investigated.

The present study investigated the effect of BP treatment on vascularization and the site-specific response of neovessel formation in jawbones and long bones to BP treatment using a skeletal mature Sprague-Dawley (SD) rat model.

## 2. Material and Methods

### 2.1. Animal Care and Grouping

Twenty-four 12-week-old female SD rats were kept in a dedicated animal holding facility under the supervision of a veterinarian in the Laboratory Animal Unit (LAU) of Li Ka Shing Faculty of Medicine, the University of Hong Kong. The animals were housed in an indoor environment at a temperature of 20°C ± 5° in a 12 : 12 h light-dark circle with free access to water and standard rodent diet (Irradiated, PMI, USA). The animal experiment was approved by the Committee on Use Live Animal for Teaching and Research, the University of Hong Kong (CULATR 3775-15).

The animals were randomly assigned into the zoledronic acid (ZA) group and control group, with twelve in each. Animals in group ZA received intraperitoneal injection (i.p.) of zoledronic acid (Zometa, Novartis, Switzerland, 0.066 mg/kg) dissolved in 0.2 ml sterile saline three times per week. This dosage scheme corresponds to 4 mg/60 kg drug dosages monthly for cancer patients with skeletal complications [5]. The control group received an equivalent amount of normal saline.

Four weeks after commencing the administration of ZA or saline, all the animals received the same procedure to create a standardized defect in the right femur and to extract the right lower and upper first molar. The animals were anesthetized with a mixture of ketamine 67 mg/kg (Alfasan International B.V., Woerden, Holland) and xylazine 6 mg/kg (Alfasan International B.V., Woerden, Holland) intraperitoneally. Using a Ø2.0 trephine bur (trephine drill 2 mm (3 mm OD) × 10 mm barrel, ForeverGreen, Hong Kong), a round defect with a diameter of 3.0 mm in the lateral aspect of the right femur was created through the cortical bone. The whole thickness of the cortical bone was removed until the marrow cavity was reached without further destroying the opposite side of the cortical bone. The right maxillary and mandibular first molars were then extracted using a standard protocol [6]. A dental explorer was used as a gingival separator to disconnect the surrounding gingiva of the molar. The right mandibular and maxillary first molars were removed using a pediatric extracting forceps.

Postoperatively, the rats were given enrofloxacin (Baytril 5%-enrofloxacin 250 ml, Bayer, Leverkusen, Germany) added in drinking water, with 2 ml in 500 ml water in q72 hours. Mobic (meloxicam, 0.6 mg/kg) and Temgesic (buprenorphine, Reckitt Benckiser Health, Slough, UK, 0.05 mg/kg) were administered subcutaneously (s/c) for pain relief. Animals were closely monitored until alert and drinking. Gel diets were given in q72 hours. The animal's clinical condition, weight, and food consumption were carefully monitored. Sutures were removed at the seventh day after the operation.

The animals were further divided into a subgroup based on the length of exposure (LoE) of drug. Four animals in the ZA group and control group were sacrificed two weeks (short term; six weeks of drug exposure), four weeks (medium term; eight weeks of drug exposure), and eight weeks (long term; twelve weeks of drug exposure) after surgery. The administration of ZA or saline continues till the sacrifice of the animals.

### 2.2. Vascularization Assessment

#### 2.2.1. Vascular Perfusion

**Figure 1:**
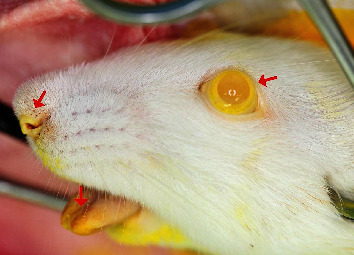
Successful perfusion is verified by turning the color of the eyes, nose, and tongue to yellow, as indicated by the arrows.

Under general anesthesia, the rat was placed supine on the operating table and the chest hair was shaved and disinfected. A midline incision was made from the sternal notch to the upper abdomen to expose the heart. The left ventricle was identified and catheterized using a 22-gauge butterfly needle, which was connected to a pressure pump set at 50 mmHg at a filling rate of 2 ml/min to replace the blood with heparinized saline (2500 U/500 ml). A small incision was made in the right atrium to facilitate draining of blood and saline water. The animal was then overdosed using the anesthetic mixture. The contrast agent—MICROFIL® MV-122 (Flow Tech, Inc., Carver, MA, USA)—was mixed (5 : 4 ratio of diluent versus compound with 5% curing agent). After rinsing the vasculature with saline solution, MICROFIL was delivered into the vasculature. Shortly after infusion of the contrast agent, the coronary arteries and ascending aorta were seen being filled with yellow contrast. Successful perfusion was checked by turning the color of the tongue, eyes, and tail to yellow (Figure 1). The animal was allowed to rest for one hour to allow for polymerizing of MICROFIL. Then, the jawbones and right femur were removed carefully without damaging the penetrating vessels. The samples were placed in 10% neutral buffered formalin solution overnight and then decalcified in 12.5% EDTA (pH = 7.2) at room temperature for three months. After decalcification was verified, samples were then transferred to 70% ethanol (ETOH) solution for micro-CT examination.

#### 2.2.2. Microcomputerized Tomography

**Figure 2:**
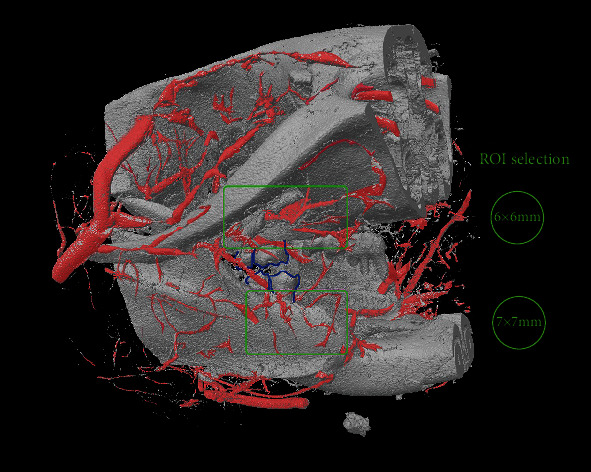
Illustration of VOI selection in the right mandible and maxilla. Vessels are labeled in pink. The right second and third molars are outlined in blue. Note that this micro-CT image is taken before decalcification; however, the analysis is done after decalcification to exclude bone tissue interference.

Plain X-ray was taken to make sure that the bone tissue was fully decalcified. The samples were then scanned with a micro-CT system (SkyScan1076; Bruker, Kontich, Belgium) following the manufacturer's instructions in the Department of Orthopedics and Traumatology, Li Ka Shing Faculty of Medicine, the University of Hong Kong. Three-dimensional images were obtained at a resolution of 8.665 *μ*m pixels. The raw dataset was reconstructed and analyzed using SkyScan CT-analyzer software (CTAn, version 1.12.0, SkyScan, Kontich, Belgium). The morphology images were primarily computed after dimensional reduction of the three-dimensional micro-CT volumes to two-dimensional sections [7, 8]. The morphometric parameters for analysis of trabecular bone microstructure were used to quantify blood vessels (Table 1) [9, 10]. The volume of interest (VOI) in the femur sample was set for 1301 layers of the 8 × 8 mm round-shape region of interest (ROI). The VOIs in the right mandible and maxilla were set for 700 layers of the 7 × 7 mm round-shape ROI and 1000 layers of the 6 × 6 mm round-shape ROI, respectively (Figure 2).

### 2.3. Statistical Analysis

All data were presented as mean ± standard deviation and statistically analyzed using two-way analysis of variance. Where applicable, further assessment of one-way analysis of variance and independent-sample *t*-test were used. Nonparametric data were analyzed using the Mann-Whitney test and Kruskal-Wallis test. *p* < 0.05 was considered statistically significant.

## 3. Results

**Figure 3:**
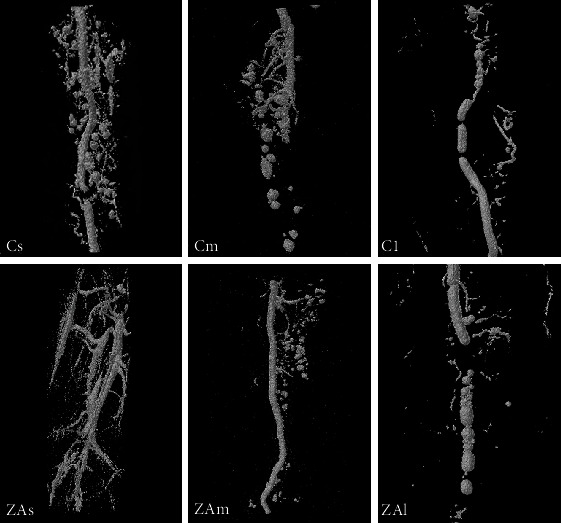
3D image of the VOI selected in the femur. Cs, Cm, and Cl: right femur of the control group at week 2, week 4, and week 8 following surgery. ZAs, ZAm, and ZAl: right femur of the ZA-treated group at week 2, week 4, and week 8 following surgery.

**Figure 4:**
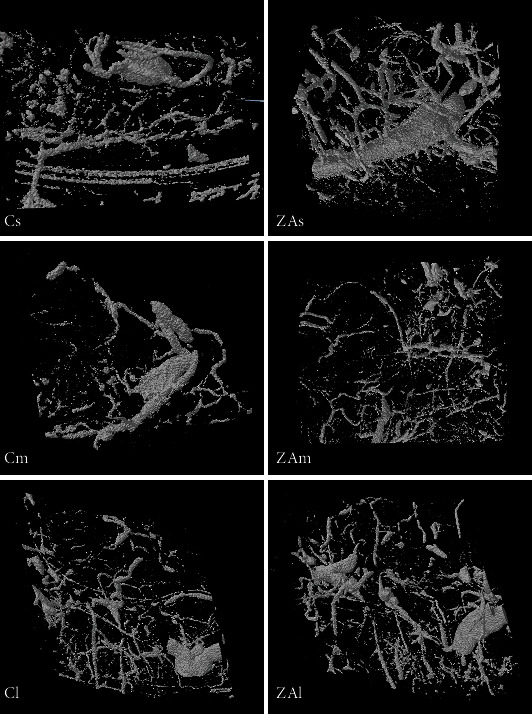
3D image of the VOI selected in the right mandible. Cs, Cm, and Cl: right mandible of the control group at week 2, week 4, and week 8 following tooth extraction. ZAs, ZAm, and ZAl: right mandible of the ZA-treated group at week 2, week 4, and week 8 following tooth extraction.

**Figure 5:**
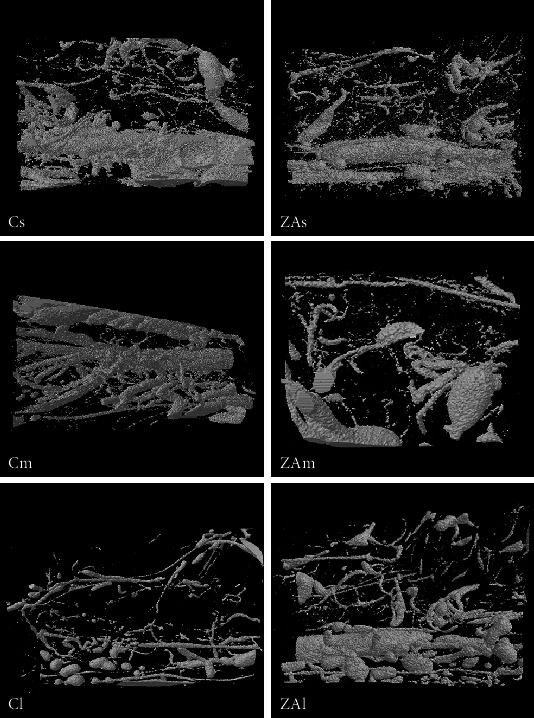
3D image of the VOI selected in the right maxilla. Cs, Cm, and Cl: right maxilla of the control group at week 2, week 4, and week 8 following tooth extraction. ZAs, ZAm, and ZAl: right maxilla of the ZA-treated group at week 2, week 4, and week 8 following tooth extraction.

Three-dimensional micro-CT assessment showed no significant change in vessel density and distribution between ZA-treated and control groups in all examined anatomical sites (Figures 3–5).

Femur: along with the healing after surgery, the branching (FD) and connectivity (V.Sp) of blood vessels and the vessel volume fraction (VVF) in the selected VOI significantly reduced.

The treatment and interaction of LoE and treatment both did not display a significant effect in any parameters in femur defect. Intergroup comparison showed that the vessel volume fraction (VVF) was higher in the short-term ZA-treated group (1.15%) compared with the control (0.58%). In medium-term and long-term groups, the VVF values in the ZA-treated sample were approximately equal to or slightly lower than that in control groups but without statistical significance.

Mandible: similar to those in the femur, the branching (FD) and connectivity (V.Sp) of blood vessels and the vessel volume fraction (VVF) in the selected VOI significantly reduced along with the healing after surgery.

Treatment of ZA did not exhibit a significant effect on vascular change in the mandibular extraction socket. Intergroup comparison showed the VVF in the ZA-treated group with lower percentage of vessel volume in all time points compared with that in the control group, yet the difference was not significant.

Maxilla: LoE was found to have a significant effect on VT, V.Sp, FD, and Eu.N (*p* < 0.05). Zoledronic acid treatment was found to significantly affect VVF and Eu.N (*p* < 0.05). Intergroup comparison showed a marginally significant higher value of the Euler number (*p* = 0.049) in the ZA-treated group in the medium-term maxilla compared with the control group. A higher Euler number was found in long-term ZA-treated maxilla, short and medium ZA-treated group comparing with their corresponding control group. However, the differences were not statistically significant.

Overall, treatment of zoledronic acid did not have significant effects on VN, VT, V.Sp, and FD value in all surgical sites, especially in the femur and mandible. The interaction of time length and treatment was not significant at any sites Tables 2–4.

## 4. Discussion

A number of studies have demonstrated that bisphosphonates inhibit angiogenesis [11, 12]. However, whether this character contributes to the development of MRONJ is still controversial. Meanwhile, the potential relationship of angiogenesis inhibition and the site-specific character of MRONJ has not been fully investigated. The existing reports concerning vascular change with BP treatment mostly come from clinical trials, in which BPs were shown to be able to reduce the thickening of arterial vessels and inhibit vascular calcification [13, 14]. The hypothesis of angiogenesis concerning MRONJ is supported by the fact that angiogenesis plays a crucial role in wound healing, and impaired wound healing of oral soft and hard tissue may lead to the formation of avascular and necrotic bone. Some studies suggested that bisphosphonates might affect blood vessel development in two ways: firstly, by high concentration of BPs in the entire circulatory system after IV BP administration and secondly, by the accumulation of local BPs in the region of wound healing where new blood vessels should develop and sprout into the wound [1, 15].

Ziebart et al. [1] showed in an *in vitro* study that human umbilical cord vein endothelial cells (HUVEC) and endothelial progenitor cells (EPC) were significantly influenced by BPs at different concentrations in cell migration ability, microvessel sprouting ability, and cell density, which might, in turn, decrease wound healing of the soft tissues and jawbones. However, the investigations on vascular changes in relation to BP treatment and the development of MRONJ are still scattered. Guevarra et al. (Guevarra, Borke et al. 2015) investigated the vascular architecture after tooth extractions in the ZA-treated rat model. Significantly thicker, less connected, and less ordered vessels were found in the ZA group compared with the control group. However, it is unclear whether the altered vessel structures are related to the development of MRONJ. Meanwhile, in femur head necrosis, BP treatment was found to improve the vascularization of bone tissue and accelerate fracture repair [16–19].

Despite these studies on angiogenesis and blood vessel structures *in vivo* and *in vitro*, no current method offers an optimal approach in angiogenesis and vascular changes in MRONJ. In the present study, vascular perfusion and 3D micro-CT analysis were used to investigate the vascular microarchitecture in the development of MRONJ.

We examined vascular anatomy at the extraction site of the mandible and maxilla and in the femur in 2, 4, and 8 weeks after surgical intervention. The experimental rats received intraperitoneal injection of zoledronic acid 0.066 mg/kg three times per week. This is a nonlethal dose corresponding to 4 mg/60 kg drug dosages monthly for cancer patients with skeletal complications. The clinical findings of MRONJ are mostly with cases of trauma, such as dental extraction. Some authors used a drilled defect rounding the dental extraction socket [20] to build up the animal model or mucosal lesion [21–24]. In the present study, we adopted simple tooth extraction to better imitate real clinical situation. The results revealed a preservation of vessel volume under ZA treatment in jawbones and long bones, with no significant changes in vessel separation, vessel number, and vessel thickness. In the mandible, although VVF in the ZA-treated mandible showed lower percentage of vessel volume in all time points compared with that in the control group, the difference was not significant. In the maxilla, which is more vascularized than the femur and mandible, our data indicates that ZA treatment significantly affect Eu.N (*p* <0.05). At all time points, the data shows that vessels are less connected and have three dimensions in the ZA-treated group than in the control group (higher Eu.N), although not significant except in the medium-term maxilla. There is another study using Wistar rats as the experimental animal with zoledronic acid administration and maxillary molar extraction, and they found a decreased blood vessel count histologically in animals with ZA treatment 7 days after dental extraction [25]. In the case of our study, the short-term group was defined to be 2 weeks after dental extraction. And our results suggest that the microvasculature structure in the surgical healing site was not significantly affected by the treatment of ZA. Wehrhan et al. [26] also reported in their study that although angiogenesis is impaired in MRONJ, vascularization remains unaffected.

In summary, micro-CT analysis indicated the microvasculature structure in the surgical healing site of jawbones and the femur was not significantly affected by the treatment of ZA in the rat model. Also, the vascular structure and angiogenesis function were not significantly affected. The angiogenesis inhibition properties of BPs may not be a direct factor in the development mechanism of MRONJ and its site-specific characteristics.

## Figures and Tables

**Table 1:** Micro-CT output parameters for the vascular perfusion sample.

Parameter	Abbreviation (unit)	Meaningfulness
Vessel volume fraction	VVF (%)	The fraction of selected VOI occupied by the volume of blood vessels
Vessel number	VN (mm^−1^)	The number of blood vessels per millimeter within the VOI
Vessel thickness	VT (mm)	The local voxel thickness within the blood vessel, which represents the average intraluminal diameter of the vessel
Vessel separation	V.Sp (mm)	The thickness of space in selected VOI, in which a higher value indicates reduced connectivity
Fractal dimension	FD	A measure of blood vessel branching
Euler number	Eu.N	A measure of blood vessel connectivity, which is calculated as Eu.N = islands − bridges (connectivity) + holes

**Table 2:** Micro-CT comparison of morphological structure of blood vessels in femoral defect in the ZA-treated group and control group.

Parameter	ZA group	Control group	ZA group	Control group	ZA group	Control group	Weeks		Treatment		Weeks *vs*. treatment	
	2 weeks		4 weeks		8 weeks		*F*	*p*	*F*	*p*	*F*	*p*
VVF (%)	1.15 ± 0.64	0.58 ± 0.17	0.23 ± 0.03	0.25 ± 0.06	0.13 ± 0.10	0.56 ± 0.52	4.131	0.046	0.039	0.847	2.446	0.132
VN (mm^−1^)	0.08 ± 0.02	0.10 ± 0.00	0.05 ± 0.00	0.08 ± 0.03	0.11 ± 0.13	0.14 ± 0.07	0.457	0.645	0.389	0.546	0.447	0.651
VT (mm)	0.17 ± 0.14	0.13 ± 0.17	0.11 ± 0.02	0.03 ± 0.01	0.02 ± 0.01	0.05 ± 0.05	1.976	0.185	0.361	0.560	0.209	0.814
V.Sp (mm)	0.52 ± 0.13	0.68 ± 0.12	0.20 ± 0.01	0.47 ± 0.40	1.66 ± 1.77	2.12 ± 1.28	4.725	0.033	0.290	0.601	0.097	0.909
FD	2.02 ± 0.26	1.71 ± 0.13	1.21 ± 0.07	1.26 ± 0.11	1.74 ± 0.41	1.51 ± 0.20	11.135	0.002	2.201	0.166	0.920	0.427
Eu.N	8,464.33 ± 1,350.91	8,726.00 ± 1,838.65	1,869.50 ± 92.63	2,537.00 ± 1,438.26	2,431.00 ± 2,508.81	2,714.50 ± 2,941.27	4.194	0.044	1.894	0.194	0.739	0.500

Differences are considered significant at p < 0.05.

**Table 3:** Micro-CT comparison of morphological structure of blood vessels in mandibular defect in the ZA-treated group and control group.

Parameter	ZA group	Control group	ZA group	Control group	ZA group	Control group	Weeks		Treatment		Weeks *vs*. treatment	
	2 weeks		4 weeks		8 weeks		*F*	*p*	*F*	*p*	*F*	*p*
VVF (%)	2.58 ± 1.63	3.81 ± 1.83	1.85 ± 0.12	2.53 ± 3.33	0.49 ± 0.69	0.43 ± 0.24	4.018	0.042	0.540	0.474	0.226	0.800
VN (mm^−1^)	0.12 ± 0.08	0.18 ± 0.13	0.02 ± 0.02	0.06 ± 0.05	0.19 ± 0.24	0.15 ± 0.05	0.612	0.556	0.435	0.520	0.775	0.480
VT (mm)	0.28 ± 0.22	0.49 ± 0.58	1.37 ± 0.45	0.68 ± 0.81	0.02 ± 0.02	0.03 ± 0.02	2.479	0.120	0.176	0.681	0.462	0.639
V.Sp (mm)	0.58 ± 0.31	0.46 ± 0.31	0.02 ± 0.00	0.45 ± 0.64	1.34 ± 0.52	2.01 ± 0.88	10.797	0.001	1.615	0.225	0.983	0.399
FD	1.88 ± 0.18	1.91 ± 0.07	2.37 ± 0.43	1.68 ± 0.37	1.31 ± 0.42	1.51 ± 0.05	10.097	0.002	1.826	0.198	5.235	0.020
Eu.N	7,688.40 ± 1,766.70	6,522.75 ± 2,553.23	8,284.67 ± 1,944.24	5,091.67 ± 2,365.49	2,954.50 ± 1,817.97	2,297.25 ± 2,964.26	3.315	0.066	3.623	0.078	3.345	0.065

Differences are considered significant at p < 0.05.

**Table 4:** Micro-CT comparison of morphological structure of blood vessels in maxillary defect in the ZA-treated group and control group.

Parameter	ZA group	Control group	ZA group	Control group	ZA group	Control group	Weeks		Treatment		Weeks *vs*. treatment	
	2 weeks		4 weeks		8 weeks		*F*	*p*	*F*	*p*	*F*	*p*
VVF (%)	2.77 ± 1.77	6.06 ± 2.28	0.66 ± 0.38	5.65 ± 4.69	1.58 ± 2.20	1.56 ± 1.48	2.831	0.095	6.134	0.028	1.538	0.251
VN (mm^−1^)	0.10 ± 0.05	0.14 ± 0.10	0.01 ± 0.00	0.02 ± 0.00	0.16 ± 0.19	0.20 ± 0.06	0.580	0.574	0.594	0.455	0.571	0.578
VT (mm)	0.38 ± 0.27	0.74 ± 0.63	0.66 ± 0.21	1.27 ± 0.76	0.07 ± 0.06	0.07 ± 0.05	4.508	0.033	0.578	0.461	0.084	0.920
V.Sp (mm)	0.40 ± 0.23	0.40 ± 0.27	0.03 ± 0.00	0.09 ± 0.04	1.06 ± 0.21	1.51 ± 0.56	18.556	0.000	1.109	0.311	0.798	0.471
FD	2.06 ± 0.33	2.10 ± 0.16	2.45 ± 0.11	1.95 ± 0.37	1.62 ± 0.25	1.64 ± 0.18	7.363	0.007	1.350	0.266	1.684	0.224
Eu.N	8,826.60 ± 1,408.50	7,519.00 ± 2,773.83	8,831.50 ± 622.96^∗^	6,543.00 ± 717.01	5,221.00 ± 1,612.20	2,825.00 ± 2,316.01	6.775	0.010	10.726	0.006	4.774	0.028

^∗^Differences are considered significant at p < 0.05.

## Data Availability

The data used to support the findings of this study are included within the article.
